# The Role, Function, and Mechanism of Long Intergenic Noncoding RNA1184 (linc01184) in Colorectal Cancer

**DOI:** 10.1155/2021/8897906

**Published:** 2021-01-29

**Authors:** Yan-Xia Sui, Dong-Li Zhao, Yan Yu, Lin-Chuan Wang

**Affiliations:** ^1^Department of Pathology, The First Affiliated Hospital of Xi'an Jiaotong University, Yan Ta Road No 277, Xi'an, Shaanxi Province 710061, China; ^2^Department of Radiotherapy, The First Affiliated Hospital of Xi'an Jiaotong University, Yan Ta Road No 277, Xi'an, Shaanxi Province 710061, China; ^3^Inspection Department of Hong-Hui Hospital, Xi'an Jiaotong University College of Medicine, Nan Guo Road No 76, Xi'an, Shaanxi Province 710041, China; ^4^Department of Clinical Laboratory, The First Affiliated Hospital of Xi'an Jiaotong University, Yan Ta Road No 277, Xi'an, Shaanxi Province 710061, China

## Abstract

**Background:**

Long intergenic noncoding RNA1184 (linc01184) has been recently discovered; however, its role in human diseases is limited to date. The present study is aimed at investigating the expression pattern and mechanism of linc01184 in colorectal cancer (CRC) tumorigenesis.

**Methods:**

The expression of linc01184 in CRC tissues and cell lines was compared with that in normal controls. The functions of linc01184 in CRC cells were identified by overexpression and small interfering RNA (siRNA) approaches in vitro. Meanwhile, the target gene prediction software, luciferase reporter, RNA pull-down, and western blotting assays were used to analyze the oncogenic mechanism.

**Results:**

We found that linc01184 was obviously upregulated in CRC tissues and cells when compared to normal controls, and its upregulation had a positive association with the CRC progression. linc01184 knockdown significantly suppressed CRC cell proliferation and invasion and promoted apoptosis. Besides, linc01184 acted as a competitive endogenous RNA (ceRNA) by directly binding to microRNA-331 (miR-331), and its overexpression resulted in notable increases of human epidermal growth factor receptor 2 (HER2), phosphorylated Ser/Thr kinases (p-Akt), and extracellular regulated protein kinase 1/2 (p-ERK1/2) at posttranscriptional levels in CRC cells, which were antagonized by miR-331.

**Conclusions:**

The findings reveal for the first time that linc01184 is an enhancer for the proliferation and invasion of CRC by functioning as a ceRNA through the linc01184-miR-331-HER2-p-Akt/ERK1/2 pathway regulatory network.

## 1. Introduction

Colorectal cancer (CRC), which is a multifactorial (such as smoking, drinking, and inadequate fiber and vitamin D intake) [1], multistep (adenocarcinoma sequence) [2] disease, is the third frequent and the second leading cause of death in cancer, accounting for approximately 1.80 million cases and 862,000 deaths in 2018 globally [3]. At present, CRC screening programs include carcinoembryonic antigen (CEA) and fecal occult blood test annually, flexible sigmoidoscopy every 5 years, or colonoscopy every 10 years for the average risk individuals at age 50 to 75 years [4–7]. However, these methods have either low sensitivity or specificity or low utilization/adherence rates; their clinical applications have been limited. Numerous regulatory noncoding RNAs (ncRNAs), such as microRNAs (miRNAs) [8–13] and long or large noncoding RNAs (lncRNAs) [14–21], have established the differential expression in CRC progression and, thus, can be used as a diagnostic tool or prognostic biomarker for CRC (Table 1).

The miRNAs (18~24 nt) can affect the expression of approximately 60% of protein-coding genes and act as negative regulators in cancers by binding the 3′ untranslated region (3′UTR) of the target mRNA [22–24]. The lncRNAs (>200 nt), which can be divided into the sense, antisense, bidirectional, intronic lncRNAs, and large intergenic ncRNAs (lincRNAs), have been implicated to interact with DNA, RNA, and protein, exerting almost all aspects of gene regulation via the molecular mechanisms of signal, decoy, guides, scaffold, and so on [24–27]. In the oncogenesis of CRC, numerous decoy lncRNAs (such as colon cancer-associated transcript-1 (MALAT1) [19], urothelial carcinoma-associated 1 (UCA1) [28], and H19 [29]) have been identified to function as competing endogenous RNAs (ceRNAs) or RNA sponges by binding miRNA and reduce the latter action on mRNA. linc01184, which is located on chromosome 5q23.3, is a newly discovered lincRNA and associated with the racial-specific erythrocyte traits [30]; however, its potential role in CRC and other cancers is still unknown. Our previous study has indicated that miR-331 is a CRC suppressor by targeting HER2 [31]. Meanwhile, we have also observed that linc01184 contains a complementary sequence for the seed region of miR-331. Thus, we perform the study to identify whether linc01184 acts as a contributor to CRC carcinogenesis by the interaction with miR-331.

In the present study, we demonstrated that linc01184 overexpression was a characteristic molecular change in CRC and had a close association with the progression of CRC. We further explored the biological roles of linc01184 in CRC cell lines in vitro and confirmed its oncogenic mechanism that linc01184 may function as a ceRNA to sponge miR-331, subsequently upregulating the expression of its target HER2 and triggering off the activation of p-Akt/p-ERK1/2 signaling pathways at the posttranscriptional levels. Thus, this study provided the first evidence that linc01184 may be a potential biomarker and play a carcinogenic role in the progression of CRC.

## 2. Materials and Methods

### 2.1. Tissue Samples and Cell Lines

Paired CRC and adjacent normal tissue samples were obtained from 247 patients (average age: 55.2 ± 10.4 years) who underwent radical resection of CRC but without any other therapeutic interventions at the First Affiliated Hospital of Xi'an Jiaotong University between 2010 and 2014. The study was approved by the Ethics Committee of the Hospital and was performed in accordance with the Declaration of Helsinki. All patients provided written informed consent before the study, and their information was kept anonymous in the study.

Samples were collected and immediately frozen in liquid nitrogen and stored at -80°C until total RNA extraction. The pathological stage was appraised by an experienced pathologist using the American Joint Committee on Cancer/Union for International Cancer Control (AJCC/UICC) tumor-node-metastasis (TNM) staging system [15]. Six CRC cell lines (HCT-116, LoVo, HT-29, SW480, DLD-1, and Caco2) and one human normal colon epithelial cell line (CRL-1831) were obtained from American Type Culture Collection (ATCC) (Manassas, VA, USA). All cells were authenticated by Beijing Genomics Institute (Shenzhen, China) based on 8 short tandem repeat (STR) loci using the ABI GeneAmp®9700 PCR system (Thermo Fisher, Waltham, MA). The cells were cultured in Dulbecco's modified Eagle's medium (DMEM) (Gibco-BRL, Gaithersburg, MD, USA) at 37°C in a humidified 5% CO_2_ incubator.

### 2.2. RNA Extraction and Real-Time Quantitative Polymerase Chain Reaction (RT-qPCR)

Total RNA from tissue samples and cells was extracted by the TRIzol reagent (Invitrogen, Carlsbad, CA, USA), and 1000 ng total RNA was converted to cDNA in a final volume of 10 *μ*L using the One-Step SuperScript III RT reagent kit (Invitrogen) following the manufacturer's protocol. The reverse transcription procedures were as follows: 45°C for 30 min and 85°C for 15 sec, and then held at 4°C. RT-qPCR was performed to determine the levels of linc01184, miR-331, and HER2 by the SYBR Green qPCR Master Mix (Thermo Fisher, Shanghai, China). All primers (Table 2) were synthesized by RiBoBio Co., Ltd. (Guangzhou, China), and U6 or *β*-actin (RiBoBio) was used as the internal reference. The RT-qPCR was conducted at 94°C for 3 min and followed by 40 cycles of 94°C for 10 sec, 55°C for 35 sec, and 55°C for 25 sec. The experiment was performed in triplicate and repeated three times; the relative levels of linc01184 and miR-331 were assessed using the 2^-*ΔΔ*Ct^ method as described previously [16].

### 2.3. Cell Transfection

The scramble control RNA and siRNA specific to linc01184 were obtained from RiBoBio. Then, the scramble control RNA and 5, 10, 20, 40, and 80 nM siRNA, respectively, were transfected into SW480 and HCT-116 cells using lipofectamine 3000 (Invitrogen) under the manufacturer's procedure.

### 2.4. Cell Proliferation Assay

The proliferation of SW480 and HCT-116 cells transfected by scramble control RNA or siRNA was assessed by the Cell Counting Kit-8 (CCK-8; Sigma). Briefly, the transfected cells (5 × 10^3^/well) were seeded into a 96-well plate (ExcellBio, Taicang, Jiangsu, China) and incubated for 24 h. Then, 10 *μ*L CCK-8 solution was added into each well and cultured for 4 h at 37°C with 5% CO_2_. Finally, the cell viability (%) was calculated on the basis of the optical densities (ODs) at 450 nm wavelength.

### 2.5. Cell Invasion Assay

The HCT-116 and SW480 cells (5 × 10^3^/well) transfected with scramble control RNA or siRNA were added to the Transwell (Corning Incorporated, NY, USA) and cultured for 48 h. After washing five times, the Transwell was placed in a 24-well plate (ExcellBio), and the cells were fixed with 95% alcohol for 10 min. After removing alcohol, the cells were stained with 0.1% crystal violet (Sigma-Aldrich, Castle Hill, NSW, Australia) for 30 min. The cell invasion was observed under an inverted microscope (Leica MZ8, Leica Microsystems, Wetzlar, Germany) at ×10 magnification.

### 2.6. Constructions of Plasmid Vectors and Luciferase Reporter Gene Vectors

Full-length linc01184 and wild-type (WT) and mutant (MUT) linc01184 were all synthesized by RiBoBio. In accordance with the manufacturer's procedure, they were cloned into pCDNA3.1 (Invitrogen) and pGL3 luciferase reporter plasmid (Promega, Madison, WI, USA) to construct the pCDNA-linc01184, luci-linc01184-WT, and luci-linc01184-MUT, respectively.

### 2.7. Dual-Luciferase Reporter Assay

The luci-linc01184-WT or luci-linc01184-MUT and miR-331 mimic or negative control (NC) mimic (RiBoBio) were cotransfected into HCT-116 cells by lipofectamine 3000 (Invitrogen), and the luciferase activity of the transfected cells was evaluated by a dual-luciferase reporter assay kit (Promega). All experiments were conducted according to the manufacturer's protocols.

### 2.8. RNA Pull-Down Assay

The biotin-labeled WT and MUT miR-331 which are obtained from Invitrogen Corporation were transfected into HCT-116 cells using lipofectamine 3000 (Invitrogen). Then, the enrichment of linc01184 in transfected cells was measured by the RNA pull-down kit (Promega) in accordance with the manufacturer's protocol.

### 2.9. Apoptosis Assay

The HCT-116 cells which are transfected with scramble control RNA, siRNA, pCDNA-linc01184, miR-331 inhibitor (RiBoBio), and siRNA+miR-331 inhibitor, respectively, were added to a 24-well plate and cultured for 48 h. The apoptosis of harvesting cells was evaluated by the FITC-Annexin V apoptosis detection kit (BD Biosciences, Piscataway, NJ, USA) according to the manufacturer's instructions.

### 2.10. Western Blotting Assay

Using the mammalian protein extraction reagent (Pierce, Rockford, IL, USA), the total proteins were extracted from HCT-116 cell lysates that are transfected with 1 *μ*g/mL pcDNA-linc01184, 20 nM siRNA, 60 nM miR-331 inhibitor, and siRNA+miR-331 inhibitor, respectively. The protein concentration was measured by a Bio-Rad protein assay kit (Bio-Rad, Hercules, CA, USA). Then, the protein was separated using 10% SDS-PAGE and transferred to a 0.22 *μ*m nitrocellulose membrane (Bio-Rad). The membrane was incubated with anti-HER2, anti-Akt, anti-p-Akt, anti-ERK1/2, and anti-p-ERK1/2 (Santa Cruz Biotechnology, Santa Cruz, CA, USA) at 4°C overnight. Then, the horseradish peroxidase- (HRP-) labeled goat anti-mouse IgG (Santa Cruz Biotechnology) was added, and the membrane was incubated for 1 h. The *β*-actin (RiBoBio) was used as the reference protein.

### 2.11. Statistical Analysis

An independent-sample *t*-test, chi-squared test, and Kruskal-Wallis rank test (one-way ANOVA) were used to analyze the data by SPSS 13.0 (serial number 5026743; SPSS Inc., Chicago, Illinois, USA). A *p* value < 0.05 (two-tailed) was considered to be statistically significant.

## 3. Results

### 3.1. linc01184 Was Highly Expressed in CRC Tissues and in Cell Lines

**Figure 1:**
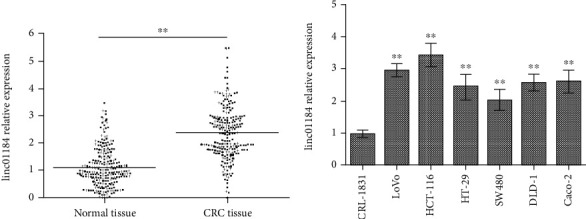
linc01184 expression in both tissues and cell lines by RT-qPCR. (a) Compared with the adjacent normal tissue, linc01184 expression was highly upregulated in colorectal cancer (CRC) tissue; (b) linc01184 expression in CRC cell lines (HCT-116, LoVo, HT-29, SW480, DLD-1, and Caco2) was significantly higher than that in a normal colon epithelial cell line (CRL-1831). ^∗∗^*p* < 0.01 vs. normal tissue or CRL-1831.

To investigate the role of linc01184 in CRC, the RT-qPCR was used to measure linc01184 expression in CRC and normal controls. We found that linc01184 expression in CRC tissues was significantly higher than that in adjacent normal tissues (2.38 ± 0.06 vs. 1.12 ± 0.05, *p* < 0.001) (Figure 1(a)). Using the median (2.43) as a cut-off, 137 CRC patients were defined as high linc01184 expression cases. Among them, linc01184 overexpression had a significant association with the tumor size, invasion for lymph node and depth, metastasis, differentiated type, and TNM stage (all *p* < 0.05) but was not significantly correlated with age, sex, and tumor location (all *p* > 0.05) (Table 3). Furthermore, linc01184 expression in six CRC cell lines was 2-3 times of that in the normal colon epithelial cell line (CRL-1831), *p* < 0.001 (Figure 1(b)). The findings indicated that linc01184 upregulation may play an oncogenic role in the progression of CRC.

### 3.2. linc01184 Knockdown Significantly Inhibited CRC Cell Proliferation and Invasion

**Figure 2:**
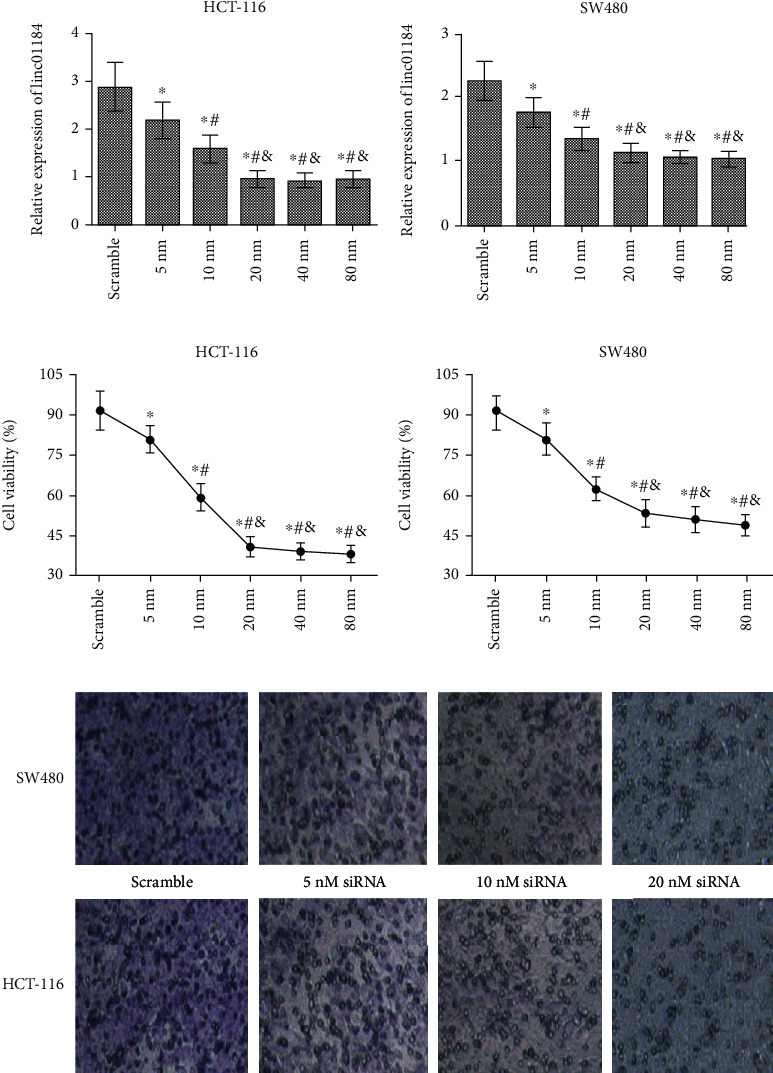
Loss of function to elucidate the effects of linc01184 on the growth of CRC cells in vitro. (a) linc01184 expression in HCT-116 and SW480 cells was significantly inhibited when transfected with siRNA; (b) CCK-8 assay showed that the cell viability in HCT-116 and SW480 cells was significantly decreased by siRNA; (c) the images under an inverted microscope showed that the invasion in HCT-116 and SW480 cells can be significantly inhibited by siRNA. ^∗^*p* < 0.05 vs. scramble control; ^#^*p* < 0.05 vs. 5 nM siRNA; ^&^*p* < 0.05 vs. 10 nM siRNA.

Furthermore, loss of function assay through RNA interference was performed to elucidate the effect of linc01184 on the CRC cells in vitro. We found that compared with the treatment of scramble control, the abilities of linc01184 expression (Figure 2(a)), cell viability (Figure 2(b)), and invasion (Figure 2(c)) in HCT-116 and SW480 cells were obviously weakened by siRNA through a dose-dependent manner. For both linc01184 expression and cell growth, the highest inhibition efficiency can be obtained at ≥20 nM siRNA. Thus, the experiments in vitro demonstrated the biological functions of linc01184 on facilitating the proliferation and invasion of CRC cells.

### 3.3. linc01184 Directly Targeted miR-331 and Acted as ceRNA in CRC Cell

**Figure 3:**
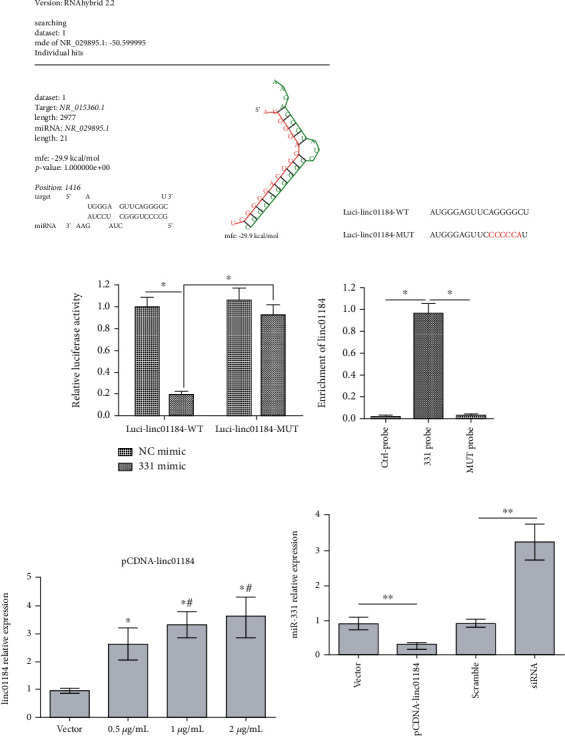
linc01184 directly targeted miR-331 and acted as ceRNA of miR-331 in CRC cells. (a) The prediction of the binding region between linc01184 and miR-331; (b) the sequence of wild-type (WT) and mutant (MUT) linc01184 luciferase reporters; (c) dual-luciferase reporter assay revealed that linc01184 directly targeted miR-331, ^∗^*p* < 0.05 vs. luci-linc01184-MUT or negative control (NC) mimic; (d) the analysis of enrichment between linc01184 and miR-331 probes using the RNA pull-down assay, ^∗^*p* < 0.05 vs. control probe or miR-331 mutant (MUT) probe; (e) linc01184 expression in transfected HCT-116 cells, ^∗^*p* < 0.05 vs. empty vector, ^#^*p* < 0.05 vs. 0.5 *μ*g/mL pCDNA-linc01184; (f) miR-331 expression in transfected HCT-116 cell, ^∗∗^*p* < 0.01 vs. empty vector or scramble control.

To verify that miR-331 (NR_029895) might be a potential target of linc01184 (NR_015360), the sequences from NCBI (https://www.ncbi.nlm.nih.gov/) were analyzed by the RNAHybrid 2.2 software. TargetScan revealed that linc01184 contains complementary sites for the seed region of miR-331 (Figure 3(a)). To further investigate the interaction between linc01184 and miR-331, the luci-linc01184-WT (AUGGGAGUUCAGGGGCU) and MUT (AUGGGAGUUCCCCCCAU) were constructed (5′⟶3′) (Figure 3(b)). Meanwhile, the luci-linc01184-WT or MUT and miR-331 mimic or NC mimic were cotransfected into HCT-116 cells; the dual-luciferase reporter assay showed that the activity of luci-linc01184-WT could be significantly inhibited by miR-331 (Figure 3(c)). Moreover, the RNA pull-down assay was conducted in HCT-116 cells transfected with miR-331-WT and MUT probes; the results revealed that linc01184 was dramatically enriched by the miR-331-WT probe (Figure 3(d)).

In addition, the RT-qPCR assay showed that the highest linc01184 expression can be obtained at ≥1 *μ*g/mL pCDNA-linc01184 transfection in HCT-116 cells, with about three times of expression compared to empty vector (Figure 3(e)). Thus, 1 *μ*g/mL pCDNA-linc01184 and 20 nM siRNA were used to evaluate the effect of linc01184 on the miR-331 expression. The data showed that linc01184 overexpression significantly repressed miR-331 expression, which was promoted when silencing linc01184 (Figure 3(f)). Our findings indicated that linc01184 directly targeted miR-331 and acted as a ceRNA by sponging miR-331.

### 3.4. linc01184 Upregulated HER2 and Inhibited Apoptosis in CRC Cell

**Figure 4:**
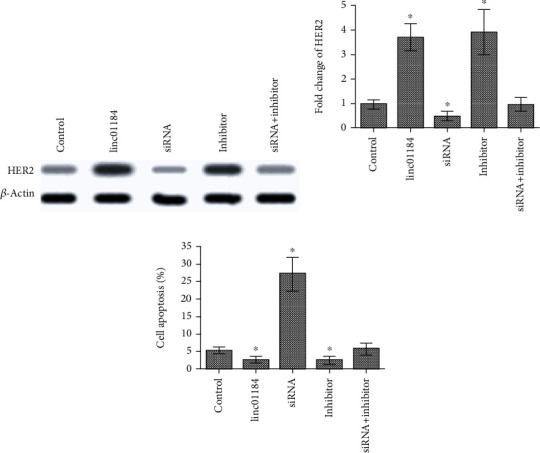
The change of HER2 protein level expression and cell apoptosis in HCT-116 cells under various conditions. (a) Western blot showed that linc01184 overexpression (pCDNA-linc01184 transfection) or miR-331 downregulation (miR-331 inhibitor transfection) significantly enhanced HER2 expression, which was abolished by siRNA; however, the effect of siRNA on HER2 expression can be antagonized by miR-331 inhibitor; (b) fold change of HER2 was significantly increased by linc01184 overexpression or miR-331 downregulation, which was opposite to siRNA transfection; (c) linc01184 overexpression or miR-331 downregulation significantly suppressed CRC cell apoptosis. ^∗^*p* < 0.05 vs. scramble control.

The target relationship between miR-331 and HER2 has been identified in our previous study [31]. Thus, to explore the effect of linc01184 on HER2 expression and apoptosis, HCT-116 cells were transfected with scramble control, 1 *μ*g/mL pCDNA-linc01184, 20 nM siRNA, 60 nM miR-331 inhibitor, and combination of siRNA and miR-331 inhibitor, respectively. We found that linc01184 overexpression or miR-331 downregulation resulted in a significant upregulation of HER2 (Figures 4(a) and 4(b)) and inhibition of apoptosis (Figure 4(c)), which were abolished by siRNA. However, the effect of siRNA on both HER2 expression and apoptosis in HCT-116 cells can be antagonized by miR-331 inhibitor (Figure 4). The results identified the functions of linc01184 on HER2 upregulation and inhibiting CRC cell apoptosis via competing with miR-331.

### 3.5. linc01184 Promoted the Akt/ERK1/2 Signaling Pathways in CRC Cell

**Figure 5:**
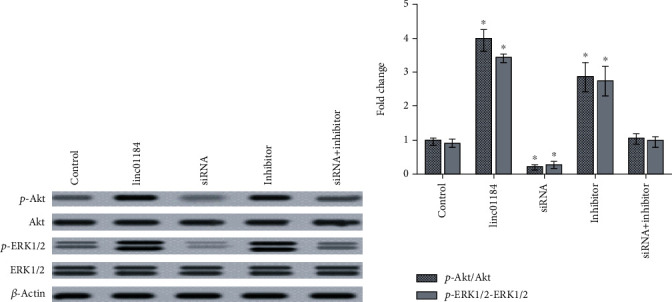
The changes of Akt and ERK1/2 protein levels in HCT-116 cells under various conditions. (a) linc01184 overexpression or miR-331 downregulation resulted in notable upregulations of p-Akt and p-ERK1/2 protein levels, which were abolished by siRNA; (b) the fold changes of p-Akt/Akt and p-ERK1/2/ERK1/2 were significantly increased by linc01184 overexpression or miR-331 downregulation. ^∗^*p* < 0.05 vs. scramble control.

To further examine whether linc01184 is involved in HER2 downstream signaling pathways, the phosphorylation levels of Ser/Thr kinases (p-Akt) and extracellular regulated protein kinase 1/2 (p-ERK1/2) under various conditions were analyzed in the present study. As presented in Figure 5(a), the protein levels of Akt and ERK1/2 are almost unchanged in HCT-116 cells transfected with 1 *μ*g/mL pCDNA-linc01184, 20 nM siRNA, 60 nM miR-331 inhibitor, or combination of siRNA and miR-331 inhibitor. However, linc01184 overexpression or miR-331 downregulation resulted in notable increases of p-Akt and p-ERK1/2 protein levels, which was opposite to siRNA transfection. The results were also been verified in Figure 5(b) that pCDNA-linc01184 or miR-331 inhibitor transfections significantly increased the ratios of both p-Akt/Akt and p-ERK1/2/ERK1/2 with respect to scramble control transfection, which were both decreased by siRNA transfection (all *p* < 0.05). Furthermore, cotransfection of siRNA and miR-331 inhibitor presented no effect on the protein levels of p-Akt and p-ERK1/2 (Figures 5(a) and 5(b)). Collectively, the results suggested that linc01184 overexpression triggered off the activation of p-Akt and p-ERK1/2 signals in CRC cells, which was antagonized by miR-331.

## 4. Discussion

The studies of human genomes have revealed that <2% of the genomes are protein-coding genes, while many noncoding elements are transcribed into ncRNAs, yielding tens of thousands of lncRNAs that lack protein-coding capacity [32–34]. Until now, only a limited number of lncRNAs are well understood for their functions; however, it is very clear that lncRNAs can play abundantly regulatory and functional roles in multiple biological processes. In cancers, lncRNAs execute their molecular functions through diverse mechanisms, such as the roles of scaffolds in chromatin remodeling [35, 36], enhancer RNA (eRNA) in chromatin interactions [37, 38], ceRNAs to sponge miRNAs [39], and natural antisense transcript (NAT) [40].

linc01184 is a newly discovered intergenic lncRNA with ~3.0 kb length, but its biological function in human diseases is limited to date. In the study, we investigated the linc01184 expression profiles in CRC, the results revealed that linc01184 was significantly upregulated in both CRC tissues and cell lines in comparison with normal controls, and its upregulation was closely associated with tumor size, invasion, metastasis, differentiation, and TNM stage of CRC. Meanwhile, siRNA-mediated linc01184 knockdown significantly inhibited CRC cell proliferation and invasion and enhanced apoptosis in vitro. These results led us to believe that linc01184 may play a carcinogenic role and is a potential biomarker in the progression of CRC.

HER2 belongs to the human epidermal growth factor receptor (EGFR) family and is an oncogenic driver to be well used as the marker of prognosis and therapeutic target in ovarian [41] and breast cancers [42]; it also plays a key factor in CRC [43]. Moreover, increasing evidences have demonstrated that lncRNAs can regulate the miRNA's expression as a ceRNA in multiple cancers, including CRC [44–46]. Our previous study has identified that miR-331 was a CRC suppressor by targeting HER2 to inhibit p-Akt/p-ERK1/2 signals [31]. Due to the expression pattern and biological function of linc01184 being opposite to those of miR-331 in CRC, thus, we hypothesized a mechanism that linc01184 may act as ceRNA to control the expression of HER2-p-Akt/p-ERK1/2 via sponging miR-331 in the development of CRC.

To validate the hypothesis of the present study, the target gene prediction software, luciferase reporter assay, and RNA pull-down assay were used to analyze the potential interactions between linc01184 and miR-331. We found that the seed sequence of miR-331 was perfectly matched with linc01184. Meanwhile, miR-331 significantly inhibited the activity of linc01184-fused luciferase and pulled down linc01184. We also found that miR-331 expression was inverse to linc01184 in CRC cells in vitro. Additionally, western blot and fold change analyses showed that linc01184 overexpression in CRC cells significantly increased protein levels of HER2, p-Akt, and p-ERK1/2, while silencing of linc01184 displayed the opposite effect. Moreover, the combined treatment of miR-331 inhibitor and siRNA-linc01184 indicated that miR-331 inhibitor could antagonize the anticancer effects of siRNA-linc01184 on cell apoptosis and deactivation of HER2/p-Akt/p-ERK1/2 signaling pathways. Therefore, the fact that linc01184 acted as ceRNA to control the expressions of HER2, p-Akt, and p-ERK1/2 at posttranscription levels via directly binding to miR-331 may be one of the potential mechanisms in CRC tumorigenesis.

## 5. Conclusions

In the current study, we identified linc01184 as a positive modulator and revealed its mechanism in CRC carcinogenesis for the first time. We found that linc01184 functioned as a ceRNA of miR-331 through the linc01184-miR-331-HER2-Akt/ERK1/2 pathway regulatory network, in which linc01184 suppressed the expression of miR-331, subsequently upregulated HER2, and stimulated the activation of Akt/ERK1/2 signals, thereby promoting CRC cell proliferation and invasion.

## Figures and Tables

**Table 1:** Regulatory ncRNAs associated with CRC.

Regulatory ncRNAs	Cytoband	Expression	Mode of action and references
*MicroRNAs (miRNAs)*			
miR-34a	1p36.22	Decreased	Regulates p53 gene expression [8]
miR-610	11p14.1	Decreased	Targets hepatoma-derived growth factor (HDGF) [9]
miR-187	18q12.2	Decreased	Targets transforming growth factor beta (TGF-*β*) [10]
miR-374a	Xq13.2	Decreased	Reducing cyclin D1 (CCND1) to inactivate the PI3K/Akt pathway [11]
miR-363-3p	Xq26.2	Decreased	Inhibits epithelial-to-mesenchymal transition (EMT) [12]
miR-15a/16	13q14.2	Decreased	Modulates the expression of genes controlling cell cycle progression [13]
*Long or large noncoding RNAs (lncRNAs)*			
Colorectal cancer-associated lncRNA (CCAL)	3q29	Elevated	Activates Wnt signaling [14]
Colon cancer-associated transcript-1-long isoform (CCAT1-L)	8q24.21	Elevated	Regulates chromatin interactions at v-MYC avian myelocytomatosis viral oncogene homolog (MYC) [15]
Colorectal neoplasia differentially expressed (CRNDE)	16q12.2	Elevated	Responds to epidermal growth factor receptor (EGFR) signaling [16]
HOX transcript antisense intergenic RNA (HOTAIR)	12q13.13	Elevated	Functions as a positive moderator of EMT [17]
lincRNA-p21	6p21.2	Decreased	Activated by p53 and modulates heterogeneous nuclear ribonucleoprotein K (hnRNP-K) [18]
Metastasis-associated lung adenocarcinoma transcript 1 (MALAT1)	11q13.1	Elevated	Promotes EMT and activates Wnt signaling [19]
Maternally expressed gene 3 (MEG3)	14q32	Decreased	Serve as p53 regulators [20]
H19	11p15.5	Elevated	Acts as precursor of miR-675 to regulate retinoblastoma gene expression and participates EMT [21]

**Table 2:** The primer sequences included in this study.

Genes	Forward primer (5′⟶3′)	Reverse primer (5′⟶3′)
Human-HER2	GGTCTTGATCCAGCGGAACC	AGCGGTTGGTGTCTATCAGT
linc01184	CACCATCGTTTGCTGAACACT	CCGCTTGCACCTTATCTCACTA
miR-331	GCCCCUGGGCCUAUCCUAGAA	Universal qPCR primer
U6	CTCGCTTCGGCAGCACA	Universal qPCR primer
*β*-Actin	CTCCATCCTGGCCTCGCTGT	GCTGTCACCTTCACCGTTCC

**Table 3:** Correlation between linc01184 expression and pathological parameters in CRC.

Parameters	No. of patients	linc01184 high expression	*χ* ^2^ value	*p* value
Age			0.158	0.691
≤60	117	62		
>60	130	75		
Sex			0.136	0.712
Male	149	80		
Female	98	57		
Tumor size			3.99	0.046^∗^
≤5 cm	171	81		
>5 cm	76	56		
Location			0.146	0.702
Colon	167	90		
Rectum	80	47		
Lymph node invasion			7.871	0.005^∗^
Negative	145	60		
Positive	102	77		
Depth of invasion			4.410	0.036^∗^
T1-T2	79	30		
T3-T4	168	107		
TNM stage			12.007	0.001^∗^
I/II	115	39		
III/IV	132	98		
Differentiated type			6.310	0.012^∗^
High/moderate	150	65		
Poor	97	72		
Metastasis			4.475	0.034^∗^
Negative	203	100		
Positive	44	37		

Note: ^∗^*p* < 0.05.

## Data Availability

The data used in the study are available from the corresponding author upon request.

## Abbreviations

ncRNA: Noncoding RNA
linc01184: Large intergenic noncoding RNA1184
CRC: Colorectal cancer
miR-331: MicroRNA-331
HER2: Human epidermal growth factor receptor 2
ceRNA: Competitive endogenous RNA
Akt: Ser/Thr kinase
ERK: Extracellular regulated protein kinases
CEA: Carcinoembryonic antigen
lncRNAs: Long noncoding RNA
UCA1: Urothelial carcinoma-associated 1
HOTAIR: HOX transcript antisense RNA
HOTTIP: Homeobox A transcript at the distal tip
GC: Gastric cancer
AJCC/UICC: American Joint Committee on Cancer/Union for International Cancer Control
DMEM: Dulbecco's modified Eagle's medium
TNM: Tumor-node-metastasis
ATCC: American Type Culture Collection
RT-qPCR: Real-time quantitative polymerase chain reaction
siRNA: Silence RNA
HRP: Horseradish peroxidase.
